# Errors in the implementation, analysis, and reporting of randomization within obesity and nutrition research: a guide to their avoidance

**DOI:** 10.1038/s41366-021-00909-z

**Published:** 2021-07-29

**Authors:** Colby J. Vorland, Andrew W. Brown, John A. Dawson, Stephanie L. Dickinson, Lilian Golzarri-Arroyo, Bridget A. Hannon, Moonseong Heo, Steven B. Heymsfield, Wasantha P. Jayawardene, Chanaka N. Kahathuduwa, Scott W. Keith, J. Michael Oakes, Carmen D. Tekwe, Lehana Thabane, David B. Allison

**Affiliations:** 1 Department of Applied Health Science, Indiana University School of Public Health-Bloomington, Bloomington, IN, USA.; 2 Department of Nutritional Sciences, Texas Tech University, Lubbock, TX, USA.; 3 Department of Epidemiology and Biostatistics, Indiana University School of Public Health-Bloomington, Bloomington, IN, USA.; 4 Division of Nutritional Sciences, University of Illinois at Urbana-Champaign, Urbana, IL, USA.; 5 Department of Public Health Sciences, Clemson University, Clemson, SC, USA.; 6 Pennington Biomedical Research Center, Louisiana State University, Baton Rouge, LA, USA.; 7 Department of Psychiatry, School of Medicine, Texas Tech University Health Sciences Center, Lubbock, TX, USA.; 8 Department of Pharmacology and Experimental Therapeutics, Division of Biostatistics, Thomas Jefferson University, Philadelphia, PA, USA.; 9 Department of Epidemiology, School of Public Health, University of Minnesota, Minneapolis, MN, USA.; 10 Department of Health Research Methods, Evidence and Impact, McMaster University, Hamilton, ON, Canada.

## Abstract

Randomization is an important tool used to establish causal inferences in studies designed to further our understanding of questions related to obesity and nutrition. To take advantage of the inferences afforded by randomization, scientific standards must be upheld during the planning, execution, analysis, and reporting of such studies. We discuss ten errors in randomized experiments from real-world examples from the literature and outline best practices for their avoidance. These ten errors include: representing nonrandom allocation as random, failing to adequately conceal allocation, not accounting for changing allocation ratios, replacing subjects in nonrandom ways, failing to account for non-independence, drawing inferences by comparing statistical significance from within-group comparisons instead of between-groups, pooling data and breaking the randomized design, failing to account for missing data, failing to report sufficient information to understand study methods, and failing to frame the causal question as testing the randomized assignment per se. We hope that these examples will aid researchers, reviewers, journal editors, and other readers to endeavor to a high standard of scientific rigor in randomized experiments within obesity and nutrition research.

## INTRODUCTION

Randomization in scientific experiments bolsters causal inference. Determining a true causal effect would require observing the difference between two outcomes within a single unit (e.g., person, animal) in one case after exogenous manipulation (e.g., “treatment”) and in another case without the manipulation, with all else, including the time of observation, held constant [1]. However, this true causal effect would require parallel universes in which the same unit at the same time undergoes manipulation in one universe but does not in the other. In the absence of parallel universes, we can estimate average causal effects by balancing all differences between multiple units, such that one group looks as similar as possible to the other group. In practice, however, balancing all variables is likely impossible. For practical application, randomization is an alternative because the selection process is independent of the individual’s pre-randomization (observed and unobserved) characteristics that could confound the outcome, and also balances in the long run the distributions of variables that would otherwise be potential confounders, thereby providing unbiased estimation of treatment effects [2]. Randomization and exogenous treatment allow inferential statistics to create unbiased effect estimates [3]. Departures from randomization may increase uncertainty and yield bias.

Randomization is a seemingly simple concept: just assign people (or more generically, “units” [e.g., mice, rats, flies, classrooms, clinics, families]) randomly to one treatment or intervention versus another. The importance of randomization may have been first recognized at the end of the nineteenth century, and formalized in the 1920s [4]. Yet since its inception there have been errors in the implementation or interpretation of randomized experiments. In 1930, the Lanarkshire Milk investigation tested whether raw or pasteurized milk altered weight and height vs. a control condition in 20,000 schoolchildren [5]. After publication of the experiment, William Gosset (writing as “Student” of “Student’s *t*-test” fame) critiqued the study [6], noting that while there was some random selection of students, a subset of the children were selected on the basis of being either “well fed or ill nourished,” which favored more of the smaller and lighter children being selected, rather than randomized, to the milk groups. Thus, the greater growth in individuals assigned to the milk groups could have been from receiving the milk intervention, or the result of selection bias, an invalidating design flaw. This violates the assumption that the intervention is independent of pre-randomization characteristics of the person being assigned.

Methodologists continue to improve our understanding of the implications of effective randomization, including random sequence generation, implementation (like allocation concealment and blinding), special randomization situations (e.g., randomizing groups of individuals), analysis (e.g., how to analyze an experiment with missing data), and reporting (e.g., how to describe the randomization procedures). Herein, we identify recent publications within obesity and nutrition literature that contain errors in these aspects (see Supplementary Table 1 for a structured list). These examples largely focus on errors arising in the context of null hypothesis significance testing;while there are misconceptions associated with the understanding of *p* values per se [7, 8], it is the framework by which authors typically draw conclusions. The examples span randomized experiments and trials, without or with control groups (i.e., randomized controlled trials [RCTs]). We use these examples to discuss how errors can bias study findings and fail to meet best practices for performing and reporting randomized studies. We clarify that the examples represent a convenience sample, and we make no claims about the frequency of these errors other than that they are frequent enough to have caught our attention. Our categories of errors are neither exhaustive nor in any rank order of severity. Furthermore, we make no assumptions about the circumstances that led to the errors. Rather, we share these examples in the spirit of Gosset who wrote in 1931 on the Lanarkshire Milk experiment, “…but what follows is written not so much in criticism of what was done…as in the hope that in any further work full advantage may be taken of the light which may be thrown on the best methods of arrangement by the defects as well as by the merits” [6].

## ERRORS IN IMPLEMENTING GROUP ALLOCATION

### 1. Error: representing nonrandom allocation methods as random

#### Description.

Participants are allocated into treatment groups by use of methods that are not random, but the study is labeled as randomized.

#### Explanation.

Allocation refers to the assignment of subjects into experimental groups. The use of random methods gives each study participant a known probability of being assigned to any experimental group. When any nonrandom allocation is used, studies should not be labeled as randomized.

#### Examples.

Authors of studies published in a sample of Chinese journals that were labeled as randomized were interviewed about their methods, and in only ~7% was randomization determined to be properly implemented [9]. Improperly labeling studies as randomized is not uncommon in both human and animal research on topics of nutrition and obesity, and can occur in different ways.

In one instance, a vitamin D supplementation trial used a nonrandomized convenience sample from a different hospital as a control group, yet labeled the trial as randomized [10]. In a reply [11], the authors suggested that no selection bias occurred during the allocation because they detected no significant differences between groups on measured covariates. However, this assumption is unjustified because (a) unobserved or mismeasured covariates can potentially introduce bias, or measurement of a covariate may be imperfect, (b) the inferential validity of randomization rests on the assumption that the distributions of all pre-randomization variables are the same in the long run across levels of the treatment groups, not that the distributions are the same across groups in any one sample, and (c) concluding that groups are identical at baseline because no significant differences were detected entails fallaciously “accepting the null.” Regardless of the lack of observed statistical differences between groups, treatment allocation was not randomized and should not be labeled as such.

In another example, researchers first allocated all participants to the intervention to ensure a sufficient sample size and then randomized future participants [12]. This violates the assumption that every subject has some probability of being assigned to every group [13]; the participants first allocated had no probability of being in the control group. In addition, those in the initial allocation wave may have had different characteristics from those with later enrollment.

If units are not all concurrently randomized (e.g., one group is enrolled at a different time), there is also a time-associated confound [14]. This is exemplified by a study of the effects of a nutraceutical formulation on hair growth that was labeled as randomized [15]. Participants were randomized to one of two treatment groups, and then each group underwent placebo and treatment sequentially (essentially a pretest-posttest design). The sequential order suggested a hair growth-by-time confound, with hair growth differing by season [16].

Nonrandom allocation can leave a signature in baseline between-group differences. With randomization, on average, the *p* values of baseline group comparisons will be uniform for independent measurements. While there are limitations to applying this principle broadly to assessing literature [17-19], in some cases it has proved useful as a prompt for more information about how and whether randomization was actually employed. An analysis by Carlisle of baseline *p* value distributions in over 5000 trials flagged apparent deviations from this expectation [20], suggesting that many studies labeled as randomized may not be. One trial flagged [21] was the Primary Prevention of Cardiovascular Disease with a Mediterranean Diet (PREDIMED) trial, which highlighted the significant impact of advice to consume a Mediterranean-style diet coupled with additional intake of extra-virgin olive oil or mixed nuts on risk for cardiovascular disease, compared with advice to consume a low-fat diet [22]. An audit by the PREDIMED authors discovered that members of some of the households were nonrandomly assigned to the same group as the randomized member. Furthermore, one intervention site switched from individuals to clinics as the randomization unit [23] (see section 5, “Error: failing to account for non-independence” for discussion of non-independence). Thus, the original analysis at the individual level was inappropriate for these participants because some did not have a known probability of being assigned to one of the treatment groups or the control. A retraction and reanalysis did not change the main results or conclusions [23], although causal language in the article was tempered. Conclusions from secondary analyses were affected, however, such as the 5-year change in body weight and waist circumference, which changed statistical significance for the olive oil group [24]. Use of statistical principles to examine the likelihood that randomization was properly implemented has flagged other studies related to nutrition and obesity, too [25-28]. In at least four cases, publications were retracted [22, 26, 29, 30].

#### Best practices.

Where randomization is impossible, methods should be clearly stated so that there is no conflation of nonrandomized with randomized experiments. Investigators should establish procedures a priori to monitor how randomization is implemented. Furthermore, although a given randomized sample may not appear balanced on all measurable baseline variables, by definition those imbalances have occurred by chance. Altering the allocation process to enforce balance with the use of nonrandom methods may introduce bias. Importantly, use of nonrandom methods may warrant changing how study results are communicated. At a practical level, most methodologists and statisticians would agree that if an RCT is properly randomized, it is reasonable to make causal claims about intervention assignment and outcomes. Whereas the purpose of most research is to seek causal effects [31], errors discussed herein break randomization, and thereby introduce additional concerns that must be satisfied to increase the confidence in unbiased estimates. While a nuanced discussion of the use of causal language is outside the scope of this review, from a purist perspective, the description of relationships as causal from nonrandom methods is inappropriate [32].

Where important pre-randomization factors are identified that could influence results if they are imbalanced (such as animal body weight), forms of restricted randomization exist to maintain the benefits of randomization with control over such factors, instead of using haphazard methods that may introduce bias. These include blocking and stratification [33, 34], which necessitate additional consideration at the analysis stage beyond a simple randomization scheme (see section 5, “Error: failing to account for non-independence”).

### 2. Error: failing to adequately conceal allocation from investigators

#### Description.

Investigators who assign treatments, and the participants receiving them, are inadequately concealed from knowing what condition was assigned.

#### Explanation.

Allocation concealment, when implemented properly, prevents researchers from foreknowing the allocation of the next participant. Furthermore, it prevents participants from foreknowing their assignment, who may choose to dropout if they do not receive a preferred treatment. Thus, concealment prevents selection bias and confounding [35-37]. Whereas randomization is a method to create unbiased estimates of effect, allocation concealment is necessary to remove the human element of decisions (whether conscious or unconscious) when participants are assigned to groups, and both are important for a rigorous trial. When concealment is broken, sample estimates can become biased in different ways.

#### Examples.

Even with the use of random allocation methods, the failure to conceal allocation means that the researchers, and sometimes participants, will know upcoming assignments. The audit of PREDIMED, as discussed in section 1, “Error: representing nonrandom allocation methods as random,” also clarified that allocation was not concealed [23], despite using computergenerated randomization tables. In the case of the Lanarkshire study as described above [5, 6], the failure to conceal allocation led to conscious bias in how schoolchildren were assigned to the interventions. In other cases, researchers may unconsciously bias allocations if they have any involvement in the allocation. For example, if the researcher who is doing the allocation is using a physical method of randomization such as rolling a die or flipping a coin in the presence of the subject, their perception of how the die or coin is rolled or flipped, or how it falls, leaves room to redo it in ways that may select for certain subjects being allocated to particular assignments.

Nonrandom allocation also may make concealment impossible; examples and explanations are presented in Table 1.

#### Best practices.

Appropriate concealment strategies may vary by study, but it is ideal that concealment be implemented. The random generation and storage of allocation codes is essential to allocation concealment, using generic numerals or letters unknown to the investigator. Electronic generation and storage of allocations in a protected centralized database is sometimes preferred [33, 38] to opaque sealed envelopes [39, 40], which is not completely immune to breach and can bias the results if poorly carried out or intentionally compromised [41-43]. Furthermore, if feasible, real-time generation may be favored over pregenerated allocations [44]. Regardless of physical or electronic concealment, the allocation codes and other important information about the assignment scheme, such as block size in permuted block randomization [45], should remain concealed from all research staff and participants. Initial allocation concealment can still be implemented and would improve the rigor of trials even if blinding (i.e., preventing post-randomization knowledge of group assignments) throughout the trial cannot be maintained.

### 3. Error: not accounting for changes in allocation ratios

#### Description.

The allocation ratio or number of treatment groups is changed partway through a study, but the change is not accounted for in the statistical analysis.

#### Explanation.

Over the course of a study, researchers may intentionally change treatment group allocations, such as adding, dropping, or combining treatment arms, for various reasons. When researchers change allocation ratios mid-study, this must be taken into account during statistical analysis [46]. Allocation ratios also change in “adaptive trials,” which have specific methods and concerns beyond what we can cover here (see [47] for more information).

#### Examples.

A study evaluating effects of weight loss on telomere length performed one phase by randomizing participants to three treatment groups (in-person counseling, telephone counseling, and usual care) with 1:1:1 allocation. After no significant difference was found between in-person and telephone counseling, participants in the next phase of the study were randomized with 1:1 allocation into a combined intervention of in-person and telephone counseling or usual care [48]. In addition to the authors’ choice of analyzing interim results before starting another phase (which risks increasing false-positive findings and should be accounted for in statistical analysis [49]), the analysis combined these two phases, effectively analyzing 2:1 and 1:1 allocations together [50]. Another study of low-calorie sweeteners and sucrose and weight-related outcomes [51] started by randomly allocating participants evenly to five treatment groups with 1:1:1:1:1 allocation, but changed to 2:1:1:1:1 midway through after one group had a higher attrition rate. Neither of these two studies reported accounting for these different phases of study in the statistical analysis. Using different allocation ratios for different groups can bias study results [46, 50]. This is because differences may exist between the different periods of recruitment in participant characteristics, such as baseline BMI [46, 50]. Thus, baseline differences in the wave of participants allocated at the 2:1 ratio, when pooled with the ratio of those allocated at the 1:1 ratio, would exaggerate the differences when analyzed as though all participants were allocated at the same time.

#### Best practices.

When allocation ratios change within studies or between randomized experiments that are pooled, caution should be used in combining data. Changes in allocation ratios must be properly taken into account in statistical analysis (see section 7, “Error: improper pooling of data”).

### 4. Error: replacements are not randomly selected

#### Description.

Participants who dropout are replaced in ways that are nonrandom, for instance, by allocating individuals to a single treatment that experienced a high percentage of participant dropout.

#### Explanation.

Nonrandom replacement of dropouts is another example of changing allocation ratios. Dropout is common in real-world studies and often leads to missing data, bias, and potentially the loss of power. A meta-analysis of pharmaceutical trials for obesity estimated an average 1-year dropout rate of 37% [52]. Similarly, a secondary analysis of a diet intervention estimated that the probability of completing the trial was only 60% after just 12 weeks [53]. Analytical approaches like intention-to-treat [ITT] analysis and imputation of data (described in the Errors in analysis section below) may obviate the need to consider replacing subjects after the initial randomization [52, 54]. Yet replacement is sometimes observed in the literature and failing to use random methods to do so introduces another source of potential bias.

In a properly implemented simple RCT, every subject will have the same a priori probability of belonging to any group as any other subject. When a subject who has dropped out is replaced with the next person enrolled instead of by using randomization for assignment, the new participant did not have the same chances as the other subjects in the study of being allocated to that group. This corrupts the process of randomization, potentially introducing bias, and compromises causal inference. Furthermore, allocating participants this way makes allocation concealment impossible.

It is vital to account for dropout in the calculation of sample size and allocation ratios when designing the study. Nevertheless, if dropout was not accounted for a priori, one option is that for the number of dropouts encountered, new participants are enrolled, but each new participant is randomly assigned to groups with the same allocation ratios as the originals [55]. Note that if dropouts are higher from a particular group and if completers only are analyzed, this may result in an imbalance in the final sample group allocation, but this is not an issue if the ITT principle is adhered to (see section 8, “Error: failing to account for missing data”).

#### Examples.

Often, studies do not specify the methods used to replace subjects and use nondescript sentences similar to “subjects who dropped out were replaced” [56-59]. As discussed in regard to a trial on green tea ointment and pain and wound healing [60], such vagueness might suggest introduction of bias and lead to questionable conclusions.

#### Best practices.

Although replacing subjects may indeed help with the problem of power, the consequences can be detrimental if not properly implemented. Therefore, the decision to replace participants should be thoroughly considered, preplanned if at all possible, and performed by using correct methods, if found to be necessary.

## ERRORS IN THE ANALYSIS OF RANDOMIZED EXPERIMENTS

### 5. Error: failing to account for non-independence

#### Description.

Groups of subjects (e.g., classrooms, schools, cages of animals) are randomly assigned to experimental conditions together but the data are analyzed as if they were randomized individually, or repeated within-subject measures are treated as independent. Or, measures are treated as independent when subjects individually randomized have repeated within-subject measures or are treated in groups.

#### Explanation.

The use of cluster randomized trial (cRCT) designs is increasing in nutrition and obesity studies, particularly for the study of school-based interventions, and in contexts where participants are exposed to the other group(s) and as such there is a lack of independence. Similarly, animals are commonly housed together (e.g., in cages, tanks) or grouped by litter. If investigators randomize all animals to treatments by groups instead of individually, this correlation must be addressed in the analysis, but is often unrecognized or ignored. These concerns also exist in cell culture experiments, for example, if treatments are randomized to an entire plate instead of individual wells. In cluster designs, the unit of randomization is the cluster, and not the individual. A frequent error in such interventions is to power and analyze the study at the individual (e.g., person, animal) level instead of the cluster level. Failing to account for within-cluster correlation (often measured by the intraclass correlation coefficient) and cluster-level impacts during study planning leads to an overestimation of statistical power [61] and typically leads to *p* values and associated confidence intervals that are artificially small [62, 63].

If cRCTs are implemented incorrectly to start, valid inferential analysis for treatment effects is not possible without untestable assumptions [61]. For instance, randomly assigning one school to an intervention and one to a control yields no degrees of freedom, akin to randomizing one individual to treatment and one to control and treating multiple measurements on each of the two individuals as though those measurements were independent [61].

Studies that randomize at the individual level may also have correlated observations that should be considered in the analysis, and so it is important to identify potential sources of clustering. For example, outcome measures may be correlated when animals are individually randomized but then group housed for treatment. Likewise, participants individually randomized may be treated in group sessions (such as classes related to the intervention), or may be grouped together within surgeons that do not equally operate in all study arms. These types of scenarios require consideration in statistical analysis [64]. When repeated measurements are taken on subjects, they similarly must account for within-subject correlation. Taking multiple measurements within individuals (e.g., measuring eyesight in the left and right eye or longitudinal data within person over time) and treating them as independent will lead to invalid inferences [64].

A distinct issue exists when using forms of restricted randomization (e.g., stratification, blocking, minimization) that are employed to have tighter control over particular factors of interest. In such situations, it is important to include the factors on which randomization restrictions occur as covariates in the statistical model to account for the added correlation between groups [65, 66]. Not doing so can result in *p* values and associated confidence intervals that are artificially large and reduced statistical power. On the other hand, given that one is likely employing restricted randomization because of a small number of units of randomization, losing even a few “denominator” degrees of freedom due to the inclusion of additional covariates in the model may also adversely affect power [67, 68].

#### Examples.

Failing to account for clustering is one of the most pervasive errors in nutrition and obesity studies that we observe [6, 61, 69-79]. A review of school-based randomized trials with weight-related outcomes found that only 21.5% of studies used intracluster correlation coefficients in their power analysis, and only 68.6% applied multilevel models to account for clustering [80]. In the most severe cases that we observe, a failure to appropriately focus on the cluster as the unit of randomization invalidates any hope of deriving causal inferences [70, 75, 81]. For additional discussion of errors in implementation and reporting in cRCTs, see ref. [61].

In an example of clustering within participants, a study of vitamin E on diabetic neuropathy randomized participants to the intervention or placebo, but for outcomes related to nerve conduction, the authors conducted measurements in limbs, stating that “left and right sides were treated independently” [82]. Because these measures were taken within the same participants, within-subject correlations must be taken into account in statistical analyses. Treating non-independent measurements as independent in statistical analysis is sometimes called “pseudoreplication” and is also a common error in animal and cell culture experiments [83].

#### Best practices.

When planning cRCTs, it is critical to perform a power calculation that incorporates the number of clusters in the design [61]. Moreover, analyses of such designs, as well as individually randomized designs, need to include the correlations from clustering for proper treatment inferences, just as repeated measurements of outcomes within subjects must be treated as non-independent.

### 6. Error: basing conclusions on within-group statistical tests instead of between-groups tests

#### Description.

Experimental groups are analyzed separately for significant differences in the change from baseline and a difference is concluded if one is significant and the other(s) not, instead of comparing directly between groups.

#### Explanation.

The probative comparison for RCTs is between groups. Sometimes, however, researchers use pre-post within-group tests and draw conclusions based on whether the within-group significance is different, for example, significant in one group but not the other (the so-called “Difference in Nominal Significance” or DINS error [84]). Using these within-group tests to imply differences between groups increases the false-positive rate of 5% for equal group sizes to up to 50% (and higher for unequal groups) [85] and is therefore invalid.

#### Examples.

The DINS error was identified in an RCT testing isomaltulose vs. sucrose in the context of effects of an energy-reduced diet on weight and fat mass, where some conclusions, such as the outcome of fat mass, were drawn from within-group comparisons but the between-group comparison was not statistically different [86]. We observe this error frequently in nutrition and obesity research [87-103]. Sometimes using this logic still reaches the correct conclusions (i.e., the between-group and within-group comparisons are both statistically significant or not), but often it does not, and therefore it is an unreliable approach for inferences.

#### Best practices.

For proper analysis of RCTs, within-group testing should not be represented as the comparison of interest [71, 84, 85, 87, 102]. Journal editors, reviewers, and readers should request that conclusions be drawn from between-group comparisons.

### 7. Error: improper pooling of data

#### Description.

Data for a single RCT are pooled without maintaining the randomized design, or data from multiple RCTs are pooled (i.e., meta-analysis) without accounting for study in statistical analysis.

#### Explanation.

Data for statistical analysis can be pooled either within one or multiple RCTs, but errors can arise when the random elements of assignment are disregarded. Pooling within one study refers to the process of combining data across different groups, subgroups, or sites to include in a single analysis. When a single RCT is performed across multiple sites or subgroups and the same allocation ratio is not used across all sites or subgroups, or the randomization allocation to study arms changes during the course of an RCT, these different sites, subgroups, or phases of the study need to be taken into account during data analysis. This is because assignment probability is confounded with subset. If data are pooled simply with no account for subsets, any differences between subsets can bias effect estimation [50].

When combining multiple RCTs, individual participant data (IPD) can be used (i.e., IPD meta-analysis). However, if they are treated as though they came from a single RCT without accounting for site, at best it will increase the residual variance and make the analysis inefficient, and at worst will confound the results and make the effect estimates biased [104]. Another error in IPD meta-analyses is the use of data pooled across trials to compare intervention effects in one subgroup of participants with another (e.g., to test the interaction between intervention and pre-randomization subgroups) without accounting for trial in the analysis. This increases the risk of bias, owing to lack of knowledge of individual within- and across-trial interaction effects and inability to separate them, as well as inappropriate standard errors for the interaction effect [105]. This differs from “typical” meta-analyses because the effect estimates already account for the fact that both treatment groups existed in the same study.

#### Examples.

In the trial of how weight loss affects telomere length in women with breast cancer (see subsection “Examples” under section 3, “Error: not accounting for changes in allocation ratios”), data were pooled from two different phases of an RCT that had different allocation ratios, which was not taken into account in the analysis [50]. Another example is a pooling study that combined IPD from multiple RCTs to examine the effects of a school-based weight management program on summer weight gain among students but ignored “study” as a factor in the analysis [106].

#### Best practices.

When pooling data under the umbrella of one study (e.g., allocation ratio change during the study), statistical analysis should include variables for subgroups to prevent confounding [46]. When pooling IPD from multiple RCTs, care must be taken to include a term for “study” when group conditions or group allocation ratios are not identical across all included RCTs [106]. For additional information on methods for IPD meta-analysis, see ref. [105].

### 8. Error: failing to account for missing data

#### Description.

Missing data (due to dropouts, errors in measurement, or other reasons) are not accounted for in an RCT.

#### Explanation.

The integrity of the randomization of subjects must be maintained throughout a study. Any post-randomization exclusion of subjects or observations, or any instances of missingness in post-randomization measurements, violates both randomization and the ITT principle (analyzing all subjects according to their original treatment assignments) and thus potentially compromises the validity of any statistical analyses and the conclusions drawn from them. There are two main reasons for this. Whereas randomization minimizes potential confounding by providing similar distributions in baseline participant characteristics, missing data that are not completely at random breaks the randomization, introduces potential bias in various ways, and degrades the confidence that the effect (or lack thereof) is the result only of the experimental condition [107, 108]. Consider as an example reported income. If individuals with very low or very high incomes are less likely to report their incomes, then nonmissing income values and their corresponding covariate values cannot provide valid inference for individuals who did not report income, because the populations are simply not the same. Missing data are extremely common in RCTs, as discussed in section 4, “Error: replacements are not randomly selected.” Regardless of the intervention, investigators need to be prepared to handle missing data based on assumptions about how data are missing.

#### Examples.

One review found that only 50% of trials use adequate methods to account for missing data [109], and studies of obesity and nutrition are no exception. For example, in a trial of intermittent vs. continuous energy restriction on body composition and resting metabolic rate with a 50% dropout rate, reanalysis of all participants halved the magnitude of effect estimates compared with analyses of completers only [99]. As in this case, investigators will often report analyses performed only on participants who have completed the study, without also reporting an ITT analysis that includes all subjects who were randomized. Investigators may dismiss ITT analyses because they perceive them as “diluting” the effect of the treatment [110]. However, this presumes that there is an effect of treatment at all. Dropouts may result in an apparent effect that is actually an artifact. If dropouts are nonrandom, then groups may simply appear different because people remaining in the treatment group are different people from those who dropped out. Attempts to estimate whether those who dropped out differ from those who stayed in are often underpowered.

Furthermore, some investigators may not understand ITT and mislabel their analysis. For instance, in an RCT of a ketogenic diet in patients with breast cancer, the authors reported that “[s] tatistical analysis was carried out according to the intention-to-treat protocol” of the 80 randomized participants, yet the flow diagram and results suggest that the analyses were restricted to completers only [111]. Surveys of ITT practices suggest that there is a general lack of adequate reporting of information pertaining to how missing data is handled [112].

#### Best practices.

Many analyses can be conducted on randomized data including “per protocol” (removing data from noncompliant subjects) and ITT. However, simply comparing per protocol to ITT analyses as a sensitivity analysis is suboptimal;they estimate different things [113]. As such, the Food and Drug Administration has recently focused on the concept of estimands to clearly establish the question being tested [114]. ITT can estimate the effect of assignment, not treatment per se, in an unbiased manner, whereas the per protocol analysis can only estimate in a way that allows the possibility for bias.

In an oft-paraphrased maxim of Lachin [108], “the best way to deal with the problem [of missing data] is to have as little missing data as possible.” This goal may be furthered through diligent administrative follow-up and constant contact with subjects; further considerations on minimization of loss-to-follow-up and other missingness may be found elsewhere [115, 116]. However, having no missing data whatsoever is often not achievable in practice, especially for large, randomized studies. Thus, something must be done when missing data exist. In general, the simplest and best way to mitigate the problem of missing data is through the ITT principle when conducting the statistical analysis.

Statistical approaches for handling missing data require untestable assumptions, assumptions that lack face validity and hence are unfounded, or both [108]. Complete case analyses, where subjects with missing data are ignored, require assumptions that the data are missing completely at random that are not recommended [108]. Multiple imputation fills in missing data repeatedly, with relationship and predictions guided by other covariates, and is recommended under the assumption that data are missing at random (MAR); that is, the missingness or not of an observation is not directly impacted by its true value. Methods commonly used in obesity trials such as last observation carried forward (LOCF) [117] or baseline observation carried forward (BOCF) are not recommended because of the strict or unreasonable assumptions required to yield valid conclusions [108, 117, 118]. In such cases where values are missing not at random (MNAR; this set of assumptions may also be referred to as “not missing at random”, NMAR), explicit modeling for the missingness process is required [119], requiring stronger assumptions that may not be valid.

Finally, when it is apparent that data are MNAR, when the integrity of randomization is no longer intact, or both, estimates are no longer represented as a causal effect afforded by randomization and care should be taken that causal language is tempered. Even in cases where the assumptions are violated, however, ignoring the missingness (e.g., completers only analyses) is generally not recommended.

In summary, minimizing missing data should be a key goal in any randomized study. But when data are missing, thoughtful approaches are necessary to respect the ITT principle and produce unbiased effect estimates. Additional discussion about best practices to handle missing data in the nutrition context is available at ref. [107].

## ERRORS IN THE REPORTING OF RANDOMIZATION

### 9. Error: failing to fully describe randomization

#### Description.

Published reports fail to provide sufficient information so that readers can assess the methods used for randomization.

#### Explanation.

Studies cannot be adequately evaluated unless methods used for randomization are reported in sufficient detail. Indeed, many examples described herein were obscured by poor reporting until we or others were able to gain clarification from the study authors through personal communication or post-publication discourse. Accepted guidelines that define the standards of reporting the results of clinical trials (i.e., Consolidated Standards of Reporting Trials for human trials (CONSORT) [120]), animal research (i.e., Animal Research: Reporting of In Vivo Experiments (ARRIVE) [121]), and others [122] have emphasized the importance of adequate reporting of randomization methods. Researchers should, to the fullest extent possible, report according to accepted guidelines as part of responsible research conduct [123].

#### Examples.

Most authors (including historically us), however, do not report adequately, and this includes randomization sequence generation and allocation concealment in human and animal research [124, 125]. We have noted specific examples of a failure to include sufficient details about the method of randomization and allocation ratio in a study of dairy- and berry-based snacks on nutritional status and grip strength [126], which were clarified in a reply [127]. In a personal communication regarding another trial of a nutritional intervention on outcomes in individuals with autism spectrum disorder, we learned that the authors had used additional blocking factors, and randomized some siblings as pairs, neither of which were reported in the paper nor accounted for in the statistical analysis [128]. In another study that pooled RCTs of school-based weight management programs, the reported number of participants of the included studies was inconsistent with the original publications [106]. In other cases, the methods used to account for clustering may not be appropriately described for readers to assess them [129, 130]. In one case, the authors reported randomizing in pairs, yet the number randomized was an odd number and differed between groups (*n* = 21 and *n* = 24) [131], to which the authors reported a coding error [132]. Other vague language descriptions include statements such as “the samples were randomly divided into two groups” [27].

The use of non-specific language to describe allocation methods may also lead to confusion as to whether randomized methods were actually used. For example, we observed the term “semi-random” used to reflect stratified randomization [133] or minimization [134], whereas elsewhere it may describe methods that are nonrandom or not clearly stated [135].

#### Best practices.

Neglecting to report essential components of how randomization was implemented hinders the ability of a reader from fully evaluating the trial and hence from interpreting the validity of the reported findings. We emphasize that reporting guidelines such as CONSORT [120] should be consulted during the study planning and publication preparation stages to ensure that essential components related to randomization are reported, such as methods used to generate the allocation sequence, implement randomization, and conceal allocation; any matching or blocking procedures used;accuracy and consistency of the numbers in flow diagrams;and reporting baseline demographic and clinical variables. With regard to the last point, a common error is to report *p* values of baseline statistical comparisons and conclude covariate imbalance between groups if they are <0.05. An example of this type of thinking is as follows: “[a]s randomization was not fully successful concerning age, it was included as covariate in the main analyses.” [136], or conversely, “The similarity between the exercise plus supplement and exercise plus placebo groups for both demographic composition and pre-intervention fitness and cognitive scores provides strong evidence that participants were randomly assigned into groups” [137]. However, as discussed in section 1, “Error: representing nonrandom allocation methods as random,” the distribution of *p* values from baseline group comparisons is uniform in the long run with randomization and therefore we would expect on average that 1/20 *p* values will be <0.05 by chance, with some caveats [17-19]. In other words, per CONSORT, “[s]uch significance tests assess the probability that observed baseline differences could have occurred by chance; however, we already know that any differences are caused by chance” [120], and should not be reported. Baseline *p* values do not reflect whether imbalances might affect the results; imbalanced variables that are prognostic on the outcome that are not *p* < 0.05 can still have a strong effect on the result [138, 139]. Thus, statistical tests should not be used to determine prognostic covariates; such covariates should preferably be identified and included in an analysis plan prior to executing the study [139].

### 10. Error: failing to properly communicate inferences from randomized studies

#### Description.

The causal question is not framed as testing the randomized *assignment* per se.

#### Explanation.

The appropriate execution and analysis of a randomized experiment tests the effect of treatment *assignment* on the outcome of interest. The causal effect being tested is what participants are assigned to, not what they actually did. That is, if some participants drop out, do not comply with the intervention, are accidentally given the wrong treatment, or in other ways do not complete the intended treatment, the proper analysis maintains the randomized *assignment* of the subjects and tests the effect of assigning subjects to the treatment, which includes factors beyond the treatment itself. Indeed, it may be that dropout or non-compliance is caused by the assignment itself. This distinction is particularly important in nutrition trials, which often suffer from poor compliance, and is discussed in part in subsection “Explanation” under section 8, “Error: failing to account for missing data” with respect to the ITT principle. For instance, researchers may be interested in discussing the effect of *eating* their diet, when in fact what was tested was being *assigned to eat* the diet.

#### Examples.

As discussed in section 8, “Error: failing to account for missing data,” there is often a perception among authors that including subjects that are, e.g., noncompliant or incorrectly assigned will preclude an understanding of the true effect of the intervention on the outcome(s) of interest. But the realization of unbiased effect estimates that the principles of randomization afford us is only achieved when subjects are analyzed as they are randomized. For example, the random assignment to 25% energy restriction of participants in a 2-year trial resulted in an average reduction of about 12% (~300 kcal) [140]. The public discussion of this trial advertised that “Cutting 300 Calories a Day Shows Health Benefits” [141]. Yet it is possible that assigning participants to cut only 300 kcal would not have produced the same benefits if they once again achieved only half of that assigned. In another example, the random assignment of high phytate bread did not lead to a statistically significant difference in whole body iron status as compared to dephytinized bread when missing data was imputed, but it was significantly higher when dropouts were excluded [98, 142, 143]. A difference cannot be concluded from these data based on the causal question of the assignment of high phytate bread, particularly because dropout was significantly higher in one group, which may create an artificial effect.

#### Best practices.

The appropriate framing of the treatment assignment (i.e., following the ITT principle) as the causal effect of interest is important when communicating and interpreting results of RCTs. From this perspective, maximizing the validity of randomized studies from planning, execution, and analysis is a matter of maintaining the randomized assignments to the greatest extent possible. To this end, randomized studies should be communicated carefully that the causal question is assignment to treatment.

## CONCLUSION

Randomization is a powerful tool to examine causal relationships in nutrition and obesity research. Empirical evidence supports the use of both randomization and allocation concealment for unbiased effect estimates. Trials with inadequate concealment are associated with larger effect estimates than are those with adequate concealment [144-147], likely reflecting bias. Despite such undesirable potential consequences, many randomized studies of humans and animals do not adequately conceal allocation [43, 124, 148]. Although more difficult to compare in human studies, the results of nonrandomized studies sometimes differ from those of randomized trials [149], while nonrandomized animal studies are associated with increased effect sizes [148]. These empirical observations are suggestive of biased estimates, and when coupled with the theoretical arguments, indicate that randomization should be implemented whenever possible. For these reasons, where randomization is implemented per the best practices described herein, the use of causal language to communicate results is appropriate. But where it is not correctly implemented or maintained, the greater potential for bias in the effect estimates and additional assumptions that need to be met to increase confidence in causal relationships invariably changes how such effects should be communicated.

Even when randomization is implemented, errors related to randomization are common, suggesting that researchers in nutrition and obesity may benefit from statistical support during the design, execution, analysis, and reporting of randomized experiments for more rigorous, reproducible, and replicable research [150]. When errors are discovered, authors and editors have a responsibility to correct the scientific record, and journals should have procedures in place to do so expeditiously [151]. The severity of the error, ranging from invalidating the conclusions [152] to simply requiring clarification, means that different considerations exist for each type of error. For example, some invalidating errors are consequent to the design and cannot be fixed, and retractions have been issued [29, 153, 154]. For other examples such as PREDIMED, for which errors in randomization required a reanalysis as a quasi-experimental design, the reanalysis, retraction, and republication serve as an important example of scientific questioning and transparency of research methods [155]. Other cases require reanalysis or reporting of the appropriate statistical analyses but are otherwise not invalidated by design flaws [88, 156]. Yet others need clarity on the methods, for instance when a study did not really use random allocation but reported as such [157].

The involvement of professional biostatisticians and others with methodological expertise from the planning stages of a study will prevent many of these errors. The use of trial and analysis plan preregistration can aid in thinking through decisions a priori while simultaneously increasing transparency and guarding against unpublished results and inflated false positives from analytic flexibility by pre-specifying outcomes and analyses [71]. Being cognizant of these errors and becoming familiar with CONSORT and other reporting guidelines enhance the value of the time, effort, and financial investment we devote to obesity and nutrition research.

## Supplementary Material

1743032_Sup_Tab

## Figures and Tables

**Table 1.** Examples of why certain allocation methods are not random and how they may break concealment.

Example of nonrandom allocation by:	How it may break randomization	How it may break concealment
Allocation of participants first to only one treatment group until desired sample size, then randomization of the rest among treatment groups (e.g., [12])	Nonrandomized participants do not have a known probability of being in the other group(s)	The researcher knows the assignments of the participants enrolled without randomization
Alternating, such as allocating every other individual (e.g., [158, 159])	Participants may enroll in groups in nonrandom ways, and with small numbers of groups this can create imbalances	The researcher knows the next group assignment
Day of the week or time of day of enrollment [160]	Participants with certain characteristics may be more likely to be available for enrollment based on the day of the week or time of day	The researcher knows the group assignment
Patient chart number [161]	Chart numbers may be associated with known or unknown patient characteristics	If chart numbers are not randomly assigned, the researcher may be able to predict the next assignment
Participant characteristics (such as in the Lanarkshire Milk study [5, 6], or matching [162, 163])	Characteristics may not be evenly distributed (i.e., confounding may occur)	The researcher may be able to predict assignments based on characteristics
