# Supplementary material for: Errors in the implementation, analysis, and reporting of randomization within obesity and nutrition research: a guide to their avoidance
Source: Int J Obes (Lond). Author manuscript; Available in PMC 2021 Nov 1. (PMC8528702; doi:10.1038/s41366-021-00909-z)
Supplement: 1743032_Sup_Tab [file NIHMS1743032-supplement-1743032_Sup_Tab.pdf]

10.1038/s41366-021-00909-z

Supplemental Table 1. Illustrative cases of errors in the implementation, analysis, and reporting of randomization in the obesity and nutrition literature

	Implementation	Best Practices	Illustrative References and Associated Discussions
1	The study is labeled as an RCT, but not all participants are randomized, or nonrandom allocation methods are used.	Label a study as randomized only when all participants were allocated using random methods. Procedures can be established *a priori* to reduce the risk that nonrandom allocation will be unintentionally employed.	• Lanarkshire Milk Investigation (nonrandom methods) (1)[a](2)[b]<br>• Nonrandomized convenience sample as control (3)[a](4)[b](5)[b]<br>• Allocated some intervention participants first (6)[ab]<br>• Carlisle's analysis of baseline p-value distributions of 5087 RCTs (7)[c]<br>• PREDIMED randomization errors (8)[a](9)[b](10)[b](11)[b](12)[b]<br>• Unlikely randomization of moxibustion and weight-related outcomes (13)[a](14)[b](15)[b]<br>• Unlikely randomization of vitamin D in patients with lupus erythematosus (16)[ab]<br>• Unlikely randomization of food service modifications and weight outcomes (17)[a](18)[b]<br>• Animal study of air pollution exposure on obesity; allocated according to baseline body weight (19)[a](20)[b]<br>• Nutraceutical formulation on hair growth; randomized within groups (21)[a](22)[b](23)[b]<br>• Supplement on cognitive function; alternating allocation used (24)[a](25)[b]<br>• Study on kiwifruit and vitamin C; some apparent nonrandom allocation (26)[a](27)[b]<br>• Unlikely randomization: **"**Patients … were randomly assigned to one of the four dietary regimens based upon dietary preferences." (28)[a](29)[b]
2	Allocation concealment is broken by using nonrandom allocation methods, or by using random methods but not adequately concealing.	Electronic or physical allocation codes should be concealed to investigators assigning participants to groups until the time of allocation.	• Supplement on cognitive function; alternating randomization used (24)[a](25)[b]<br>• Lanarkshire Milk Investigation (nonrandom methods) (1)[a](2)[b]<br>• PREDIMED randomization errors (8)[a](9)[b](10)[b](11)[b](12)[b]<br>• Surgical comparisons for the treatment of obesity; hospital chart number used to allocate (nonrandom methods) (30)[a]<br>• Iron supplementation and anemia during pregnancy; randomized by day of week (nonrandom methods) (31)[a]
3	Different allocation ratios are used within a study but are not accounted for in the analysis.	Changing allocation ratios within or between experiments must be accounted for in statistical analysis.	• Weight loss on telomere length; allocation ratio changed in second phase of study (32)[a](33)[b]<br>• Low-calorie sweeteners and sucrose on weight-related outcomes; allocation ratio changed after higher attrition rate in one group (34)[a]
4	Participants who drop out are replaced in a way that is unclear or nonrandom.	Replacing participants in a randomized design must use random allocation.	• Green tea ointment on pain and wound healing; nonrandom replacement (35)[a](36)[b](37)[b]<br>• Other various nutrition- and obesity-related studies using vague language about replacement (38-40)[a]
	**Analysis**	**Best Practices**	**Illustrative References and Associated Discussions**
5	Non-independence is not accounted for in the design and/or analysis in randomized trials.	The planning and analysis of group-randomized designs must account for the number of groups and correlations among participants for valid inferences.	• Various nutrition- and obesity-related studies not accounting for clustering in the design and/or analysis (41-43); (44, 45); (41, 43, 46); (44, 45); (47); (48-50); (51-53); (54-57); (58, 59); (60); (61); (62, 63); (64, 65); (66-68); (69); (70); (1, 2)<br>• Review of failure to account for clustering in school-based randomized trials (71)[c]<br>• Reviews of best practices and additional discussion of clustering errors (72, 73)[c]<br>• Vitamin E on diabetic neuropathy; non-independent within-person measurements (69)[a]

6	Conclusions are drawn from within-group tests instead of between-group in RCTs.	The proper analysis of RCTs is a direct between-group test.	• Isomaltulose vs. sucrose on weight-related outcomes; some conclusions drawn from within-group comparisons (74)[a](75)[b](76)[b](77)[b]<br>• Other various nutrition- and obesity-related studies drawing conclusions from within-group instead of between-group comparisons (78, 79); (80-82); (83, 84); (85, 86); (87, 88); (89, 90); (91-93); (94, 95); (96-99); (100-102); (103-105); (106-108); (109, 110); (111)<br>• Reviews on best practices and additional discussion of these errors (73, 112-116)[c]
7	Pooled data does not account for random assignment in the analysis.	Pooling data within one or multiple studies requires consideration in the statistical analysis.	• School-based weight management program on weight gain; combined multiple RCTs without accounting for study (117)[a](118)[b]
8	Missing data is not accounted for in the analysis.	Following the ITT principle when conducting statistical analysis is the recommended approach to maintain the integrity of randomization.	• Intermittent vs. continuous energy restriction on body composition and metabolic rate; missing data ignored (100)[a](101)[b](102)[b]<br>• High phytate vs. dephytinized bread on iron status; missing data ignored (96)[a](97)[b](98)[b](99)[b]
	**Reporting**	**Best Practices**	**Illustrative References and Associated Discussions**
9	The methods used to randomize are insufficiently or incorrectly reported and may lead to confusion, or in fact do not describe random methods.	Fully report the methods used to randomize subjects by following appropriate reporting guidelines.	• Dairy- and berry-based snacks on nutritional status and grip strength; insufficient details on randomization method and allocation ratio (119)[a](120)[b](121)[b]<br>• Nutritional intervention on outcomes in individuals with autism spectrum disorder; unreported blocking factors and clustering (41)[a](46)[b](43)[b]<br>• School-based weight management program on weight gain; participant numbers inconsistent with original publications (117)[a](118)[b]<br>• Classroom intervention on diabetes control; participant number was odd yet randomized in pairs (122)[a](123)[b]<br>• Intervention to increase physical activity in fathers and daughters; whether clustering in analysis was account for unclear (124)[a](125)[b](126)[b]<br>• Breakfast on appetite and satiety; 'semi-random' used for stratified randomization (127)[a]<br>• Sucralose on metabolic sensitivity to sugar; 'semi-random' used for minimization (128)[a]<br>• Kiwifruit and vitamin C on vitality; unreported allocation methods (26)[a](27)[b]
10	Failing to properly communicate inferences.	Frame the treatment *assignment* as the causal effect of interest.	• Energy restriction on metabolic outcomes; random assignment is the exposure, not the level of restriction (129)[a](130)[b]

[a] Denotes reference where error occurred
[b] Denotes post-publication discussion or correction of error
[c] Denotes review/tutorial/original research paper about error

References

1. Leighton G, McKinlay PL. Milk Consumption and the Growth of School Children. Report on an Investigation in Lanarkshire Schools: SCOTLAND., Edinburgh : H.M.S.O.; 1930. 20 pp. p.
2. Student. The Lanarkshire Milk Experiment. Biometrika. 1931;23(3/4):398-406.
3. Rostami M, Tehrani FR, Simbar M, Bidhendi Yarandi R, Minooee S, Hollis BW, et al. Effectiveness of prenatal vitamin D deficiency screening and treatment program: a stratified randomized field trial. The Journal of Clinical Endocrinology & Metabolism. 2018;103(8):2936-48.
4. Shub A, McCarthy EA. Letter to the Editor:"Effectiveness of Prenatal Vitamin D Deficiency Screening and Treatment Program: A Stratified Randomized Field Trial". The Journal of Clinical Endocrinology & Metabolism. 2018;104(2):337-8.
5. Ramezani Tehrani F, Minooee S, Rostami M, Bidhendi Yarandi R, Hosseinpanah F. Response to Letter to the Editor:"Effectiveness of Prenatal Vitamin D Deficiency Screening and Treatment Program: A Stratified Randomized Field Trial". The Journal of Clinical Endocrinology & Metabolism. 2018;104(2):339-40.
6. Williams LK, Abbott G, Thornton LE, Worsley A, Ball K, Crawford D. Improving perceptions of healthy food affordability: results from a pilot intervention. International Journal of Behavioral Nutrition and Physical Activity. 2014;11(1):33.
7. Carlisle JB. Data fabrication and other reasons for non-random sampling in 5087 randomised, controlled trials in anaesthetic and general medical journals. Anaesthesia. 2017;72(8):944-52.
8. Estruch R, Ros E, Salas-Salvadó J, Covas M-I, Corella D, Arós F, et al. Primary prevention of cardiovascular disease with a Mediterranean diet. New England Journal of Medicine. 2013;368(14):1279-90.
9. The Editors Of The Lancet Diabetes E. Retraction and republication-Effect of a high-fat Mediterranean diet on bodyweight and waist circumference: a prespecified secondary outcomes analysis of the PREDIMED randomised controlled trial. Lancet Diabetes Endocrinol. 2019;7(5):334.
10. Estruch R, Ros E, Salas-Salvado J, Covas MI, Corella D, Aros F, et al. Primary Prevention of Cardiovascular Disease with a Mediterranean Diet Supplemented with Extra-Virgin Olive Oil or Nuts. N Engl J Med. 2018;378(25):e34.
11. Estruch R, Martínez-González MA, Corella D, Salas-Salvadó J, Fitó M, Chiva-Blanch G, et al. Effect of a high-fat Mediterranean diet on bodyweight and waist circumference: a prespecified secondary outcomes analysis of the PREDIMED randomised controlled trial. The Lancet Diabetes & Endocrinology. 2019;7(5):e6-e17.
12. Estruch R, Ros E, Salas-Salvadó J, Covas M-I, Corella D, Arós F, et al. Retraction and republication: primary prevention of cardiovascular disease with a Mediterranean diet. N Engl J Med 2013; 368: 1279-90. Mass Medical Soc; 2018.
13. Hsieh C-H, Tseng C-C, Shen J-Y, Chuang P-Y. Randomized controlled trial testing weight loss and abdominal obesity outcomes of moxibustion. Biomedical engineering online. 2018;17(2):1-10.
14. Mestre LM, Dickinson SL, Golzarri-Arroyo L, Brown AW, Allison DB. Data anomalies and apparent reporting errors in 'Randomized controlled trial testing weight loss and abdominal obesity outcomes of moxibustion'. BioMedical Engineering OnLine. 2020;19(1):1-3.
15. Hsieh C-H, Tseng C-C, Shen J-Y, Chuang P-Y. Retraction Note to: Randomized controlled trial testing weight loss and abdominal obesity outcomes of moxibustion. BioMedical Engineering OnLine. 2020;19(1):1-.
16. Abou-Raya A, Abou-Raya S, Helmii M. The effect of vitamin D supplementation on inflammatory and hemostatic markers and disease activity in patients with systemic lupus erythematosus: a randomized placebo-controlled trial. The Journal of rheumatology. 2013;40(3):265-72.
17. Hosseini R, Mirghotbi M, Pourvali K, Kimiagar SM, Rashidkhani B, Mirghotbi T. The effect of food service system modifications on staff body mass index in an industrial organization. Journal of Paramedical Sciences (JPS). 2015;6(1):2008-4978.

18. George BJ, Brown AW, Allison DB. Errors in statistical analysis and questionable randomization lead to unreliable conclusions. J Paramed Sci. 2015;6(3):153-4.
19. Wei Y, Zhang J, Li Z, Gow A, Chung KF, Hu M, et al. Chronic exposure to air pollution particles increases the risk of obesity and metabolic syndrome: findings from a natural experiment in Beijing. The FASEB Journal. 2016;30(6):2115-22.
20. Zhang J, Wei Y, Allison DB. Comment on: "Chronic exposure to air pollution particles increases the risk of obesity and metabolic syndrome: findings from a natural experiment in Beijing" [Available from: https://hypothes.is/a/AQKsEg1lEeiymitN4n0bQQ.
21. Tenore GC, Caruso D, Buonomo G, D'Avino M, Santamaria R, Irace C, et al. Annurca Apple Nutraceutical Formulation Enhances Keratin Expression in a Human Model of Skin and Promotes Hair Growth and Tropism in a Randomized Clinical Trial. Journal of medicinal food. 2018;21(1):90-103.
22. Keith SW, Brown AW, Heo M, Heymsfield SB, Allison DB. Re:“Annurca Apple Nutraceutical Formulation Enhances Keratin Expression in a Human Model of Skin and Promotes Hair Growth and Tropism in a Randomized Clinical Trial” by Tenore et al.(J Med Food 2018; 21: 90–103). Journal of medicinal food. 2019;22(12):1301-2.
23. Tenore GC, Caruso D, Buonomo G, D'Avino M, Santamaria R, Irace C, et al. Response to Keith et al. Re:“Annurca Apple Nutraceutical Formulation Enhances Keratin Expression in a Human Model of Skin and Promotes Hair Growth and Tropism in a Randomized Clinical Trial”. Journal of medicinal food. 2019;22(12):1303-4.
24. Ito N, Saito H, Seki S, Ueda F, Asada T. Effects of composite supplement containing astaxanthin and sesamin on cognitive functions in people with mild cognitive impairment: a randomized, double-blind, placebo-controlled trial. Journal of Alzheimer's Disease. 2018;62(4):1767-75.
25. Hannon BA, Oakes JM, Allison DB. Alternating Assignment was Incorrectly Labeled as Randomization. Journal of Alzheimer's Disease. 2019.
26. Conner TS, Fletcher BD, Pullar JM, Spencer E, Mainvil LA, Vissers M. KiwiC for Vitality: Results of a Randomized Placebo-Controlled Trial Testing the Effects of Kiwifruit or Vitamin C Tablets on Vitality in Adults with Low Vitamin C Levels. Nutrients. 2020;12(9):2898.
27. Vorland CJ, Jamshidi-Naeini Y, Golzarri-Arroyo L, Brown AW, Allison D. Mixed Random and Nonrandom Allocation, and Group Randomization Have Been Mislabeled and Misanalysed, Necessitating Reanalysis. Comment On:“KiwiC for Vitality: Results of a Randomized Placebo-Controlled Trial Testing the Effects of Kiwifruit or Vitamin C Tablets on Vitality in Adults with Low Vitamin C Levels”. 2021.
28. Fleming RM. The effect of high-, moderate-, and low-fat diets on weight loss and cardiovascular disease risk factors. Preventive cardiology. 2002;5(3):110-203.
29. Taubes G. Dietary Fat, Cont'd.: The Washington Post; 2002 [Available from: https://www.washingtonpost.com/archive/lifestyle/wellness/2002/09/24/interactions/dfcd1470-eedb-474f-8ae8-714836e810d9/.
30. Griffen Jr WO, Young VL, Stevenson CC. A prospective comparison of gastric and jejunoileal bypass procedures for morbid obesity. Surgery for Obesity and Related Diseases. 2005;1(2):163-72.
31. Rae P, Robb P. Megaloblastic anaemia of pregnancy: a clinical and laboratory study with particular reference to the total and labile serum folate levels. Journal of Clinical Pathology. 1970;23(5):379-91.
32. Sanft T, Usiskin I, Harrigan M, Cartmel B, Lu L, Li F-Y, et al. Randomized controlled trial of weight loss versus usual care on telomere length in women with breast cancer: the lifestyle, exercise, and nutrition (LEAN) study. Breast cancer research and treatment. 2018;172(1):105-12.
33. Dickinson SL, Golzarri-Arroyo L, Brown AW, McComb B, Kahathuduwa CN, Allison DB. Change in study randomization allocation needs to be included in statistical analysis: comment on ‘Randomized controlled trial of weight loss versus usual care on telomere length in women with breast cancer: the lifestyle, exercise, and nutrition (LEAN) study’. Breast cancer research and treatment. 2019;175(1):263-4.

34. Higgins KA, Mattes RD. A randomized controlled trial contrasting the effects of 4 low-calorie sweeteners and sucrose on body weight in adults with overweight or obesity. The American journal of clinical nutrition. 2019;109(5):1288-301.
35. Shahrahmani H, Kariman N, Jannesari S, Rafieian-Kopaei M, Mirzaei M, Ghalandari S, et al. The effect of green tea ointment on episiotomy pain and wound healing in primiparous women: A randomized, double-blind, placebo-controlled clinical trial. Phytotherapy research. 2018;32(3):522-30.
36. Golzarri-Arroyo L, Dickinson SL, Allison DB. Replacement of dropouts may bias results: Comment on" The effect of green tea ointment on episiotomy pain and wound healing in primiparous women: A randomized, double-blind, placebo-controlled clinical trial". Phytotherapy research: PTR. 2019.
37. Kariman N, Nia HS, Shahrahmani H, Ghadicolaei HT, Gorji MAH, Bagheri B, et al. In Reply to the editor: Replacement of dropouts may bias result: comment on "The effect of green tea ointment on episiotomy pain and wound healing in primiparous women: A randomized, double-blind, placebo-controlled clinical trial". Pain. 2020;3(4.52):1.523.
38. Lichtenstein AH, Jalbert SM, Adlercreutz H, Goldin BR, Rasmussen H, Schaefer EJ, et al. Lipoprotein response to diets high in soy or animal protein with and without isoflavones in moderately hypercholesterolemic subjects. Arteriosclerosis, thrombosis, and vascular biology. 2002;22(11):1852-8.
39. Draijer R, de Graaf Y, Slettenaar M, de Groot E, Wright C. Consumption of a polyphenol-rich grape-wine extract lowers ambulatory blood pressure in mildly hypertensive subjects. Nutrients. 2015;7(5):3138-53.
40. de Clercq NC, van den Ende T, Prodan A, Hemke R, Davids M, Pedersen HK, et al. Fecal Microbiota Transplantation from Overweight or Obese Donors in Cachectic Patients with Advanced Gastroesophageal Cancer: A Randomized, Double-blind, Placebo-controlled, Phase II Study. Clinical Cancer Research. 2021.
41. Adams JB, Audhya T, Geis E, Gehn E, Fimbres V, Pollard EL, et al. Comprehensive Nutritional and Dietary Intervention for Autism Spectrum Disorder-A Randomized, Controlled 12-Month Trial. Nutrients. 2018;10(3).
42. Vorland CJ, Brown AW, Dickinson SL, Gelman A, Allison DB. Comment on: Comprehensive Nutritional and Dietary Intervention for Autism Spectrum Disorder—A Randomized, Controlled 12-Month Trial, Nutrients 2018, 10, 369. Nutrients. 2019;11(5):1126.
43. Adams JB, Audhya T, Geis E, Gehn E, Fimbres V, Pollard EL, et al. Reply to "Comment on Comprehensive Nutritional and Dietary Intervention for Autism Spectrum Disorder—A Randomized, Controlled 12-Month Trial, Nutrients 2018, 10, 369". Nutrients. 2019;11(5):1138.
44. Koretz RL. JPEN Journal Club 45. Cluster Randomization. Journal of Parenteral and Enteral Nutrition. 2019;43(7):941-3.
45. Müller I, Schindler C, Adams L, Endes K, Gall S, Gerber M, et al. Effect of a multidimensional physical activity intervention on body mass index, skinfolds and fitness in South African children: results from a cluster-randomised controlled trial. International journal of environmental research and public health. 2019;16(2):232.
46. Vorland CJ, Brown AW, Dickinson SL, Gelman A, Allison DB. The Implementation of Randomization Requires Corrected Analyses. Comment on "Comprehensive Nutritional and Dietary Intervention for Autism Spectrum Disorder—A Randomized, Controlled 12-Month Trial, Nutrients 2018, 10, 369". Nutrients. 2019;11(5):1126.
47. Meurer ST, Lopes ACS, Almeida FA, Mendonça RdD, Benedetti TRB. Effectiveness of the VAMOS Strategy for Increasing Physical Activity and Healthy Dietary Habits: a randomized controlled community trial. Health Education & Behavior. 2019;46(3):406-16.
48. Aceves-Martins M, Llauradó E, Tarro L, Moriña D, Papell-Garcia I, Prades-Tena J, et al. A school-based, peer-led, social marketing intervention to engage Spanish adolescents in a healthy lifestyle ("We Are cool"—som la pera study): A parallel-cluster randomized controlled study. Childhood Obesity. 2017;13(4):300-13.
49. Aceves-Martins M, Llauradó E, Tarro L, Moriña D, Papell-Garcia I, Prades-Tena J, et al. Response to: Comment About Statistical Analysis of a Cluster-Randomized Trial About Clustering and Nesting (DOI: 10.1089/chi. 2019.0142). Childhood Obesity. 2020;16(2):67-9.

50. Golzarri-Arroyo L, Oakes JM, Brown AW, Allison DB. Incorrect analyses of cluster-randomized trials that do not take clustering and nesting into account likely lead to p-values that are too small. Childhood Obesity. 2020.
51. Kobel S, Wirt T, Schreiber A, Kesztyüs D, Kettner S, Erkelenz N, et al. Intervention effects of a school-based health promotion programme on obesity related behavioural outcomes. Journal of obesity. 2014;2014.
52. Kobel S, Wirt T, Schreiber A, Kesztyüs D, Kettner S, Erkelenz N, et al. Response to: Comment on "Intervention effects of a school-based health promotion programme on obesity related behavioural outcomes". Journal of obesity. 2015;2015.
53. Li P, Brown AW, Oakes JM, Allison DB. Comment on "Intervention Effects of a School-Based Health Promotion Programme on Obesity Related Behavioural Outcomes". J Obes. 2015;2015:708181.
54. Kobel S, Wartha O, Lämmle C, Dreyhaupt J, Steinacker JM. Intervention effects of a kindergarten-based health promotion programme on obesity related behavioural outcomes and BMI percentiles. Preventive Medicine Reports. 2019;15:100931.
55. Vorland CJ, Brown AW, Kahathuduwa CN, Dawson JA, Gletsu-Miller N, Kyle TK, et al. Questions on 'Intervention effects of a kindergarten-based health promotion programme on obesity related behavioural outcomes and BMI percentiles'. Preventive Medicine Reports. 2020;17.
56. Kobel S, Dreyhaupt J, Wartha O, Lämmle C, Steinacker JM. Response to "Questions on "Intervention effects of a kindergarten-based health promotion programme on obesity related behavioural outcomes and BMI percentiles"" by Vorland et al. Preventive Medicine Reports. 2020;17.
57. Vorland CJ, Brown AW, Kahathuduwa CN, Dawson JA, Gletsu-Miller N, Kyle TK, et al. Comment On: "Intervention effects of a kindergarten-based health promotion programme on obesity related behavioural outcomes and BMI percentiles" 2019 [Available from: https://pubpeer.com/publications/3F9C696A4855C9626FE7F805C2082C.
58. Coknaz D, Mirzeoglu AD, Atasoy HI, Alkoy S, Coknaz H, Goral K. A digital movement in the world of inactive children: favourable outcomes of playing active video games in a pilot randomized trial. European journal of pediatrics. 2019;178(10):1567-76.
59. Golzarri-Arroyo L, Vorland CJ, Thabane L, Oakes JM, Hunt ET, Brown AW, et al. Incorrect design and analysis render conclusion unsubstantiated: comment on "A digital movement in the world of inactive children: favourable outcomes of playing active video games in a pilot randomized trial". European Journal of Pediatrics. 2020:1-2.
60. Marwaha RK, Mithal A, Bhari N, Sethuraman G, Gupta S, Shukla M, et al. Supplementation with Three Different Daily Doses of Vitamin D 3 in Healthy Pre-pubertal School Girls: A Cluster Randomized Trial. Indian Pediatrics. 2018;55(11):951-6.
61. Hivner EA, Hoke AM, Francis EB, Lehman EB, Hwang GW, Kraschnewski JL. Training teachers to implement physical activity: Applying social cognitive theory. Health Education Journal. 2019;78(4):464-75.
62. Short KR, Chadwick JQ, Cannady TK, Branam DE, Wharton DF, Tullier MA, et al. Using financial incentives to promote physical activity in American Indian adolescents: A randomized controlled trial. PloS one. 2018;13(6):e0198390.
63. Golzarri-Arroyo L, Chen X, Dickinson SL, Short KR, Thompson DM, Allison DB. Corrected analysis of 'Using financial incentives to promote physical activity in American Indian adolescents: A randomized controlled trial'confirms conclusions. Plos one. 2020;15(6):e0233273.
64. Kain J, Concha F, Moreno L, Leyton B. School-based obesity prevention intervention in Chilean children: effective in controlling, but not reducing obesity. Journal of obesity. 2014;2014.
65. Li P, Brown AW, Oakes JM, Allison DB. Comment on "School-Based Obesity Prevention Intervention in Chilean Children: Effective in Controlling, but not Reducing Obesity". J Obes. 2015;2015:183528.
66. Scherr RE, Linnell JD, Dharmar M, Beccarelli LM, Bergman JJ, Briggs M, et al. A multicomponent, school-based intervention, the shaping healthy choices program, improves nutrition-related outcomes. Journal of nutrition education and behavior. 2017;49(5):368-79. e1.
67. Wood AC, Brown AW, Li P, Oakes JM, Pavela G, Thomas DM, et al. A Comment on Scherr et al "A Multicomponent, School-Based Intervention, the Shaping Healthy Choices Program, Improves Nutrition-Related Outcomes". J Nutr Educ Behav. 2018;50(3):324-5.

68. Scherr RE, Linnell JD, Dharmar M, Beccarelli LM, Bergman JJ, Briggs M, et al. Response to "A Comment on Scherr et al 'A Multicomponent, School-Based Intervention, the Shaping Healthy Choices Program, Improves Nutrition-Related Outcomes'". Journal of nutrition education and behavior. 2018;50(3):326-7.
69. Ng YT, Phang SCW, Tan GCJ, Ng EY, Botross Henien NP, M Palanisamy UD, et al. The Effects of Tocotrienol-Rich Vitamin E (Tocovid) on Diabetic Neuropathy: A Phase II Randomized Controlled Trial. Nutrients. 2020;12(5):1522.
70. Mietus-Snyder M, Narayanan N, Krauss RM, Laine-Graves K, McCann JC, Shigenaga MK, et al. Randomized nutrient bar supplementation improves exercise-associated changes in plasma metabolome in adolescents and adult family members at cardiometabolic risk. PloS one. 2020;15(10):e0240437.
71. Heo M, Nair SR, Wylie-Rosett J, Faith MS, Pietrobelli A, Glassman NR, et al. Trial Characteristics and Appropriateness of Statistical Methods Applied for Design and Analysis of Randomized School-Based Studies Addressing Weight-Related Issues: A Literature Review. J Obes. 2018;2018:8767315.
72. Brown AW, Li P, Bohan Brown MM, Kaiser KA, Keith SW, Oakes JM, et al. Best (but oft-forgotten) practices: designing, analyzing, and reporting cluster randomized controlled trials. Am J Clin Nutr. 2015;102(2):241-8.
73. Brown AW, Altman DG, Baranowski T, Bland JM, Dawson JA, Dhurandhar NV, et al. Childhood obesity intervention studies: A narrative review and guide for investigators, authors, editors, reviewers, journalists, and readers to guard against exaggerated effectiveness claims. Obesity Reviews. 2019.
74. Lightowler H, Schweitzer L, Theis S, Henry CJ. Changes in Weight and Substrate Oxidation in Overweight Adults Following Isomaltulose Intake During a 12-Week Weight Loss Intervention: A Randomized, Double-Blind, Controlled Trial. Nutrients. 2019;11(10):2367.
75. Vorland CJ, Kyle TK, Brown AW. Comparisons of Within-Group Instead of Between-Group Affect the Conclusions. Comment on:"Changes in Weight and Substrate Oxidation in Overweight Adults Following Isomaltulose Intake during a 12-Week Weight Loss Intervention: A Randomized, Double-Blind, Controlled Trial". Nutrients 2019, 11 (10), 2367. Nutrients. 2020;12(8):2335.
76. Lightowler H, Schweitzer L, Theis S, Henry CJ. Reply to "Comparisons of Within-Group Instead of Between-Group Affect the Conclusions. Comment on: Changes in Weight and Substrate Oxidation in Overweight Adults Following Isomaltulose Intake during a 12-Week Weight Loss Intervention: A Randomized, Double-Blind, Controlled Trial. Nutrients 2019, 11 (10), 2367". Nutrients. 2020;12(8):2339.
77. PubPeer Comment On: Changes in Weight and Substrate Oxidation in Overweight Adults Following Isomaltulose Intake During a 12-Week Weight Loss Intervention: A Randomized, Double-Blind, Controlled Trial. 2020.
78. Vijayakumar V, Shankar NR, Mavathur R, Mooventhan A, Anju S, Manjunath N. Diet enriched with fresh coconut decreases blood glucose levels and body weight in normal adults. Journal of Complementary and Integrative Medicine. 2018;15(3).
79. Kroeger CM, Brown AW, Allison DB. Differences in Nominal Significance (DINS) Error leads to invalid conclusions: Letter regarding,"Diet enriched with fresh coconut decreases blood glucose levels and body weight in normal adults". Journal of Complementary and Integrative Medicine. 2019;16(2).
80. Wewalka M, Drolz A, Seeland B, Schneeweiss M, Schmid M, Schneeweiss B, et al. Different enteral nutrition formulas have no effect on glucose homeostasis but on diet-induced thermogenesis in critically ill medical patients: a randomized controlled trial. European journal of clinical nutrition. 2018;72(4):496-503.
81. Koretz RL. JPEN Journal Club 40. Differences in Nominal Significance. JPEN Journal of parenteral and enteral nutrition. 2019;43(2):311.
82. Dickinson SL, Brown AW, Mehta T, Heymsfield SB, Ebbeling CB, Ludwig DS, et al. Incorrect analyses were used in "Different enteral nutrition formulas have no effect on glucose homeostasis but on diet-induced thermogenesis in critically ill medical patients: a randomized controlled trial" and corrected analyses are requested. European journal of clinical nutrition. 2019;73(1):152-3.
83. Fidanci BE, Akbayrak N, Arslan F. Assessment of a health promotion model on obese Turkish children. journal of nursing research. 2017;25(6):436-46.

84. Brown AW, Allison DB. Letter to the Editor And response Letter to the Editor and Author Response of Assessment of a Health Promotion Model on Obese Turkish Children. The Journal of Nursing Research, 25(6), 436-446. J Nurs Res. 2018;26(5):373-4.
85. Zhao J, Dong J-N, Gong J-F, Wang H-G, Li Y, Zhang L, et al. Impact of enteral nutrition on energy metabolism in patients with Crohn's disease. World Journal of Gastroenterology: WJG. 2015;21(4):1299.
86. Kaiser KA, George BJ, Allison DB. Re: Errors in Zhao et al (2015), Impact of enteral nutrition on energy metabolism in patients with Crohn's disease. World journal of gastroenterology. 2016;22(9):2867.
87. Dawson JA, Brown AW, Allison DB. The Stated Conclusions Are Contradicted by the Data, Based on Inappropriate Statistics, and Should Be Corrected: Comment on "Intervention for childhood obesity based on parents only or parents and child compared with follow-up alone". Pediatric obesity. 2018;13(11):656.
88. Yackobovitch-Gavan M, Wolf Linhard D, Nagelberg N, Poraz I, Shalitin S, Phillip M, et al. Intervention for childhood obesity based on parents only or parents and child compared with follow-up alone. Pediatric obesity. 2018;13(11):647-55.
89. Rshikesan P, Subramanya P, Singh D. Sleep quality and body composition variations in obese male adults after 14 weeks of yoga intervention: A randomized controlled trial. International journal of yoga. 2017;10(3):128.
90. Allison D. The Conclusions Are Unsupported by the Data, Are Based on Invalid Analyses, Are Incorrect, and Should be Corrected: Letter Regarding "Sleep Quality and Body Composition Variations in Obese Male Adults after 14 weeks of Yoga Intervention: A Randomized Controlled Trial". International journal of yoga. 2018;11(1):83-4.
91. Dimova RB, Allison DB. Inappropriate statistical method in a parallel-group randomized controlled trial results in unsubstantiated conclusions. Nutrition journal. 2015;15(1):58.
92. Cassani RSL, Fassini PG, Silvah JH, Lima CMM, Marchini JS. Retraction Note: Impact of weight loss diet associated with flaxseed on inflammatory markers in men with cardiovascular risk factors: a clinical study. Nutrition journal. 2015;15(1):1-.
93. Cassani RSL, Fassini PG, Silvah JH, Lima CMM, Marchini JS. Impact of weight loss diet associated with flaxseed on inflammatory markers in men with cardiovascular risk factors: a clinical study. Nutrition journal. 2015;14(1):1-8.
94. Panayotov VS. Studying a Possible Placebo Effect of an Imaginary Low-Calorie Diet. Frontiers in psychiatry. 2019;10:550.
95. Dickinson SL, Foote G, Allison DB. Commentary: Studying a Possible Placebo Effect of an Imaginary Low-Calorie Diet. Frontiers in Psychiatry. 2020;11:329.
96. Hoppe M, Ross AB, Svelander C, Sandberg A-S, Hulthén L. Low-phytate wholegrain bread instead of high-phytate wholegrain bread in a total diet context did not improve iron status of healthy Swedish females: a 12-week, randomized, parallel-design intervention study. European journal of nutrition. 2019;58(2):853-64.
97. Vorland CJ, Mestre LM, Mendis SS, Brown AW. Within-group comparisons led to unsubstantiated conclusions in "Low-phytate wholegrain bread instead of high-phytate wholegrain bread in a total diet context did not improve iron status of healthy Swedish females: a 12-week, randomized, parallel-design intervention Study". Eur J Nutr. 2020.
98. Hoppe M, Ross AB, Svelander C, Sandberg AS, Hulthen L. Reply to the comments by Vorland et al. on our paper: "low-phytate wholegrain bread instead of high-phytate wholegrain bread in a total diet context did not improve iron status of healthy Swedish females: a 12-week, randomized, parallel-design intervention study". Eur J Nutr. 2020.
99. Hoppe M, Ross AB, Svelander C, Sandberg AS, Hulthen L. Correction to: Low-phytate wholegrain bread instead of high-phytate wholegrain bread in a total diet context did not improve iron status of healthy Swedish females: a 12 week, randomized, parallel-design intervention study. Eur J Nutr. 2020.
100. Campbell BI, Aguilar D, Colenso-Semple LM, Hartke K, Fleming AR, Fox CD, et al. Intermittent Energy Restriction Attenuates the Loss of Fat Free Mass in Resistance Trained Individuals. A Randomized Controlled Trial. Journal of Functional Morphology and Kinesiology. 2020;5(1):19.

101. Peos J, Brown AW, Vorland CJ, Allison DB, Sainsbury A. Contrary to the Conclusions Stated in the Paper, Only Dry Fat-Free Mass Was Different between Groups upon Reanalysis. Comment on:“Intermittent Energy Restriction Attenuates the Loss of Fat-Free Mass in Resistance Trained Individuals. A Randomized Controlled Trial”. Journal of Functional Morphology and Kinesiology. 2020;5(4):85.
102. Campbell BI. A Reply to Contrary to the Conclusions Stated in the Paper, Only Dry Fat-Free Mass Was Different between Groups upon Reanalysis—Comment on:“Intermittent Energy Restriction Attenuates the Loss of Fat-Free Mass in Resistance Trained Individuals: A Randomized Controlled Trial”. Journal of Functional Morphology and Kinesiology. 2020;5(4):86.
103. de Sousa Canheta AB, de Souza JD, Silveira EA. Traditional Brazilian diet and extra virgin olive oil reduce symptoms of anxiety and depression in individuals with severe obesity: Randomized clinical trial. Clinical Nutrition. 2021;40(2):404-11.
104. Eckert I. Letter to the editor: Inadequate statistical inferences in the randomized clinical trial by Canheta et al. Clinical Nutrition. 2021;40(1):338.
105. de Sousa Canheta AB, de Souza JD, Silveira EA. Adequate statistical analyses and inferences of Randomized Clinical Trial. Clinical Nutrition. 2021.
106. Vorland CJ, Foote G, Dickinson SL, Mayo-Wilson E, Allison DB, Brown AW. Letter to the Editor Medicine Correspondence Blog2020 [Available from: https://journals.lww.com/md-journal/Blog/MedicineCorrespondenceBlog/pages/post.aspx?PostID=126.
107. Abdelbasset WK, Tantawy SA, Kamel DM, Alqahtani BA, Elnegamy TE, Soliman GS, et al. Effects of high-intensity interval and moderate-intensity continuous aerobic exercise on diabetic obese patients with nonalcoholic fatty liver disease: A comparative randomized controlled trial. Medicine. 2020;99(10):e19471.
108. Erratum. Medicine. 2020;99(37):e22388.
109. Aiso I, Inoue H, Seiyama Y, Kuwano T. Compared with the intake of commercial vegetable juice, the intake of fresh fruit and komatsuna (Brassica rapa L. var. perviridis) juice mixture reduces serum cholesterol in middle-aged men: a randomized controlled pilot study. Lipids in health and disease. 2014;13(1):1-8.
110. Allison DB, Antoine LH, George BJ. Incorrect statistical method in parallel-groups RCT led to unsubstantiated conclusions. Lipids in health and disease. 2016;15(1):1-5.
111. Vargas-Molina S, Carbone L, Romance R, Petro JL, Schoenfeld BJ, Kreider RB, et al. Effects of a low-carbohydrate ketogenic diet on health parameters in resistance-trained women. European Journal of Applied Physiology. 2021:1-11.
112. Bland JM, Altman DG. Comparisons against baseline within randomised groups are often used and can be highly misleading. Trials. 2011;12(1):264.
113. Bland JM, Altman DG. Best (but oft forgotten) practices: testing for treatment effects in randomized trials by separate analyses of changes from baseline in each group is a misleading approach. Am J Clin Nutr. 2015;102(5):991-4.
114. Allison DB, Brown AW, George BJ, Kaiser KA. Reproducibility: A tragedy of errors. Nature. 2016;530(7588):27-9.
115. Sainani K. Misleading comparisons: the fallacy of comparing statistical significance. PM&R. 2010;2(6):559-62.
116. George BJ, Beasley TM, Brown AW, Dawson J, Dimova R, Divers J, et al. Common scientific and statistical errors in obesity research. Obesity (Silver Spring). 2016;24(4):781-90.
117. Reesor L, Moreno JP, Johnston CA, Hernandez DC. School-Based Weight Management Program Curbs Summer Weight Gain Among Low-Income Hispanic Middle School Students. Journal of School Health. 2019;89(1):59-67.
118. Jayawardene WP, Brown AW, Dawson JA, Kahathuduwa CN, McComb B, Allison DB. Conditioning on" study" is essential for valid inference when combining individual data from multiple randomized controlled trials: a comment on Reesor et al's School-based weight management program curbs summer weight gain among low-income Hispanic middle school students. J Sch Health. 2019; 89 (1): 59-67. The Journal of school health. 2019;89(7):515.

119. Nykänen I, Törrönen R, Schwab U. Dairy-Based and Energy-Enriched Berry-Based Snacks Improve or Maintain Nutritional and Functional Status in Older People in Home Care. The journal of nutrition, health & aging. 2018;22(10):1205-10.
120. Kahathuduwa CN, Allison DB. Letter to the editor: Insufficient Reporting of Randomization Procedures and Unexplained Unequal Allocation: A Commentary on "Dairy-Based and Energy-Enriched Berry-Based Snacks Improve or Maintain Nutritional and Functional Status in Older People in Home Care. J Nutr Health Aging. 2019;23(4):396.
121. Nykänen I. Insufficient Reporting of Randomization Procedures and Unexplained Unequal Allocation: A Commentary on "Dairy-Based and Energy-Enriched Berry-Based Snacks Improve or Maintain Nutritional and Functional Status in Older People in Home Care". The journal of nutrition, health & aging. 2019;23(4):397-.
122. Barnard ND, Levin SM, Gloede L, Flores R. Turning the waiting room into a classroom: weekly classes using a vegan or a portion-controlled eating plan improve diabetes control in a randomized translational study. Journal of the Academy of Nutrition and Dietetics. 2018;118(6):1072-9.
123. Erratum. J Acad Nutr Diet. 2019;119(8):1391-3.
124. Morgan PJ, Young MD, Barnes AT, Eather N, Pollock ER, Lubans DR. Engaging fathers to increase physical activity in girls: the "dads and daughters exercising and empowered"(DADEE) randomized controlled trial. Annals of Behavioral Medicine. 2019;53(1):39-52.
125. Tekwe CD, Allison DB. Randomization by Cluster, But Analysis by Individual Without Accommodating Clustering in the Analysis Is Incorrect: Comment. Annals of Behavioral Medicine. 2019.
126. Morgan PJ, Young MD, Barnes AT, Eather N, Pollock ER, Lubans DR. Correction That the Analyses Were Adjusted for Clustering: A Response to Tekwe et al. Annals of Behavioral Medicine. 2020.
127. Douglas SM, Byers AW, Leidy HJ. Habitual Breakfast Patterns Do Not Influence Appetite and Satiety Responses in Normal vs. High-Protein Breakfasts in Overweight Adolescent Girls. Nutrients. 2019;11(6):1223.
128. Dalenberg JR, Patel BP, Denis R, Veldhuizen MG, Nakamura Y, Vinke PC, et al. Short-Term Consumption of Sucralose with, but Not without, Carbohydrate Impairs Neural and Metabolic Sensitivity to Sugar in Humans. Cell Metab. 2020;31(3):493-502 e7.
129. Kraus WE, Bhapkar M, Huffman KM, Pieper CF, Das SK, Redman LM, et al. 2 years of calorie restriction and cardiometabolic risk (CALERIE): exploratory outcomes of a multicentre, phase 2, randomised controlled trial. The lancet Diabetes & endocrinology. 2019;7(9):673-83.
130. O'Connor A. Cutting 300 Calories a Day Shows Health Benefits 2019 [Available from: https://www.nytimes.com/2019/07/16/well/eat/cutting-300-calories-a-day-shows-health-benefits.html.
